# Supplementary material for: The Gut Bacteria Dysbiosis Contributes to Chronic Graft-Versus-Host Disease Associated With a Treg/Th1 Ratio Imbalance
Source: Front Microbiol. 2022 Sep 8;13:813576. doi: 10.3389/fmicb.2022.813576 (PMC9493085; doi:10.3389/fmicb.2022.813576)
Supplement: Supplementary file 1 [file Data_Sheet_1.zip › P101SC18090073-01-B1-3-4_result/04.BetaDiversity/PCoA/unweighted_unifrac/PCoA12-2.pdf]

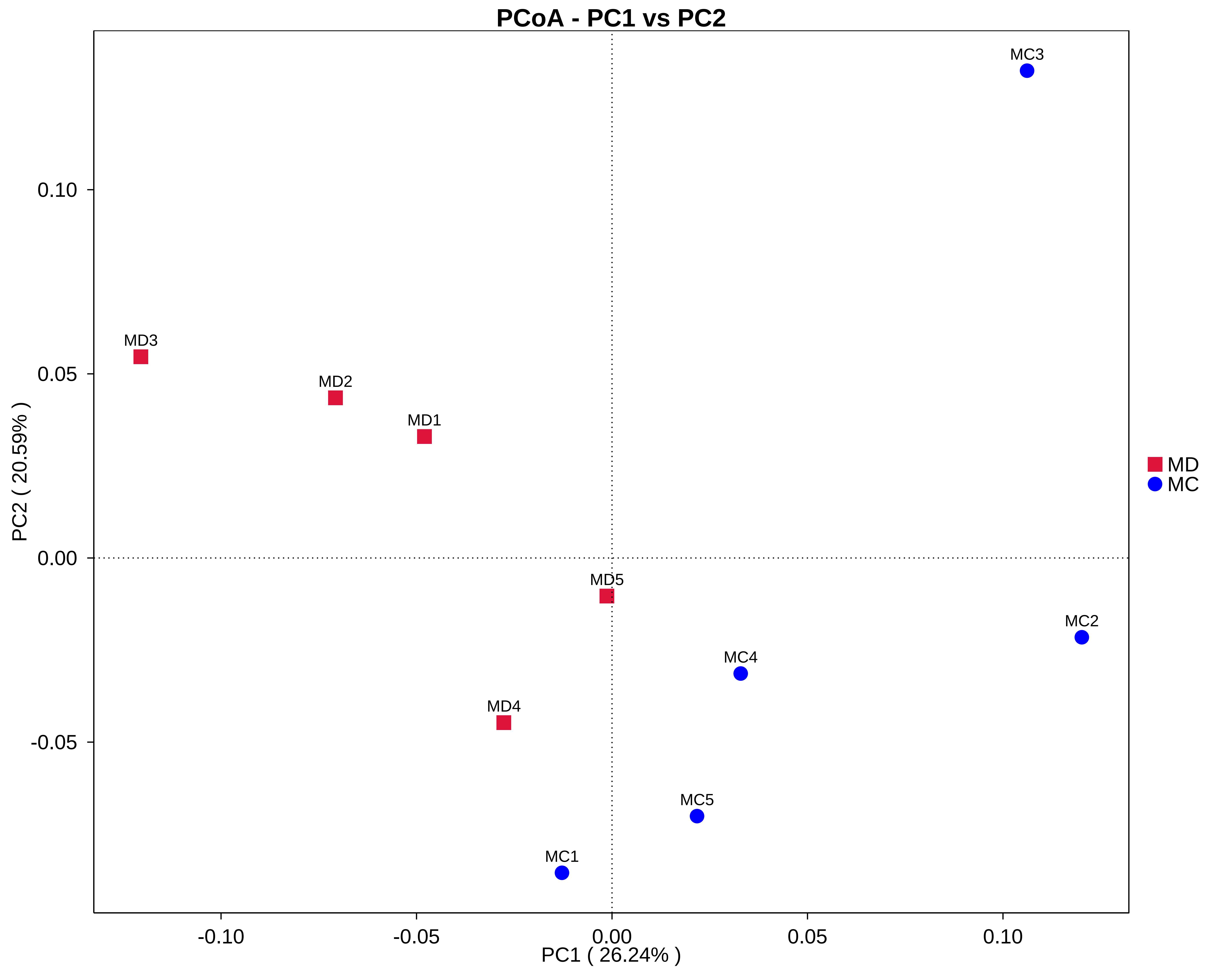
PCoA - PC1 vs PC2
PC2 (20.59%)
PC1 (26.24%)
0.10
0.05
0.00
-0.05
-0.10
-0.05
0.00
0.05
0.10
MC3
MD3
MD2
MD1
MD5
MC2
MC4
MD4
MC5
MC1
MD
MC
